# Current treatment options for cluster headache: limitations and the unmet need for better and specific treatments—a consensus article

**DOI:** 10.1186/s10194-023-01660-8

**Published:** 2023-09-04

**Authors:** Nunu Laura Timotheussen Lund, Anja Sofie Petersen, Rolf Fronczek, Jacob Tfelt-Hansen, Andrea Carmine Belin, Tore Meisingset, Erling Tronvik, Anna Steinberg, Charly Gaul, Rigmor Højland Jensen

**Affiliations:** 1 Danish Headache Center, Department of Neurology, Rigshospitalet-Glostrup, Valdemar Hansens Vej 5, 2600 Glostrup, Denmark; 2 Department of Neurology, Sjællands Universitetshospital Roskilde, Roskilde, Denmark; 3 Department of Neurology, Leiden University Medical Centre, Leiden, The Netherlands; 4 Stichting Epilepsie Instellingen Nederlands (SEIN), Sleep-Wake Centre, Heemstede, The Netherlands; 5 Department of Cardiology, Copenhagen University Hospital - Rigshospitalet, Copenhagen, Denmark; 6 Department of Forensic Medicine, Faculty of Health and Medical Sciences, Copenhagen, Denmark; 7 Centre for Cluster Headache, Department of Neuroscience, Karolinska Institutet, Stockholm, Sweden; 8 Norwegian Advisory Unit On Headaches, St. Olav University Hospital, Trondheim, Norway; 9 NorHEAD, Norwegian Headache Research Centre, NTNU, Trondheim, Norway; 10 Department of Clinical Neuroscience, Karolinska Institutet, Stockholm, Sweden; 11 Department of Neurology, Karolinska University Hospital, Stockholm, Sweden; 12 Charly Gaul, Headache Center, Frankfurt, Germany

**Keywords:** Cluster headache, Treatment, Burden, CGRP, Verapamil, Review

## Abstract

**Aim:**

Treatment for cluster headache is currently based on a trial-and-error approach. The available preventive treatment is unspecific and based on few and small studies not adhering to modern standards. Therefore, the authors collaborated to discuss acute and preventive treatment in cluster headache, addressing the unmet need of safe and tolerable preventive medication from the perspectives of people with cluster headache and society, headache specialist and cardiologist.

**Findings:**

The impact of cluster headache on personal life is substantial. Mean annual direct and indirect costs of cluster headache are more than 11,000 Euros per patient. For acute treatment, the main problems are treatment response, availability, costs and, for triptans, contraindications and the maximum use allowed. Intermediate treatment with steroids and greater occipital nerve blocks are effective but cannot be used continuously. Preventive treatment is sparsely studied and overall limited by relatively low efficacy and side effects. Neurostimulation is a relevant option for treatment-refractory chronic patients. From a cardiologist’s perspective use of verapamil and triptans may be worrisome and regular follow-up is essential when using verapamil and lithium.

**Conclusion:**

We find that there is a great and unmet need to pursue novel and targeted preventive modalities to suppress the horrific pain attacks for people with cluster headache.

**Graphical Abstract:**

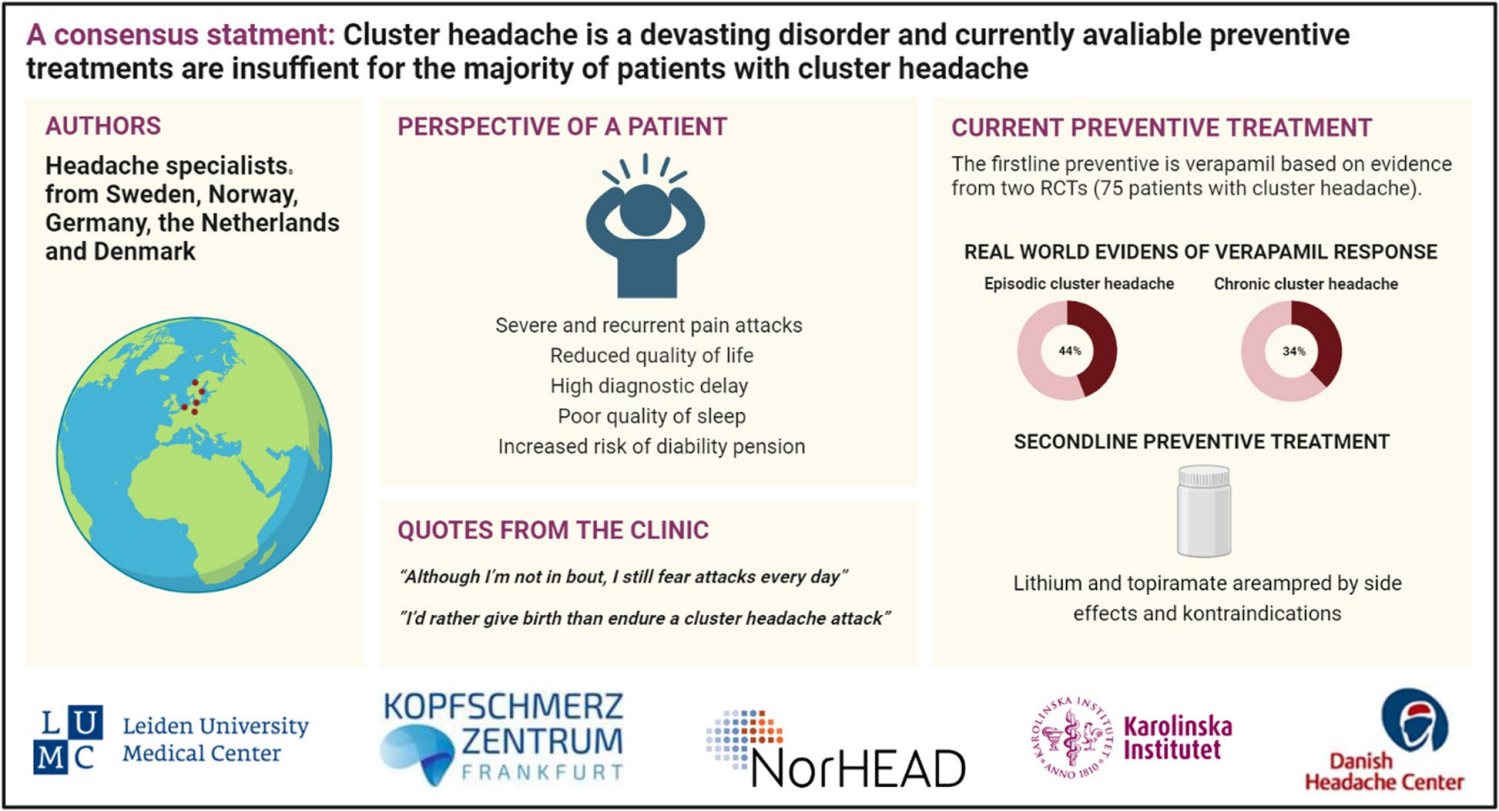

## Introduction

Cluster headache (CH) is the most common trigemino-autonomic cephalalgia [1]. The recurrent attacks up to 8 times per day are among the most severe pains described by humans, succeeding gun-shot wounds, giving birth and kidney stones [2]. The life-time prevalence is 1,24/1000 [3] and the typical age of onset is 20–40 years [4].

Existing treatments for CH have originally been developed for other medical conditions and are based on empirical data [5–8]. The three existing European guidelines for the management of CH are based on very few and small studies mostly not fulfilling modern standards: The 2023 European Academy of Neurology Guidelines on Cluster Headache, the 2006 European Federation of Neurological Societies (EFNS) guidelines on the treatment of CH and other Trigemino-autonomic cephalalgias (mainly for Neurologists) and the European Headache Federations (EHF) guidelines for headache disorders (mainly for Primary Care Physicians) [9–11]. In addition, national guidelines exist.

Therefore, the aim of this paper is to provide insights into the unmet need for safe and tolerable CH preventive medication from the perspective of people with CH and society, headache specialist and cardiologist. To do this, we review and discuss existing treatment possibilities for CH. Neurostimulation and future perspectives are also discussed in this consensus paper arising from some of the major CH clinics and research centers in Europe.

### From the perspective of patient and society

”I’d rather give birth than endure a cluster headache attack”

CH has an impact on all aspects of peoples’ lives including higher proportions of multimorbidity of somatic and psychiatric diseases [12–14]. In a recent interview-based Danish study on personal and economic burden, 92% of people with episodic CH (ECH) in bout, 98% of people with chronic CH (CCH) and even 15% people with ECH in remission reported to be restricted in their everyday lives [15]. People with CH do not present with any physical handicaps, hindering understanding from family, friends and colleagues [16]. Overall, the disease mainly affects the younger half of the population where careers and family lives are being established, and in 21% and 48% of people with ECH and CCH, CH led to dependency on family and friends [15]. In clinical experience, family members report feeling helpless and afraid because people with CH may get irritable or aggressive as part of their attacks or might even become self-harming.

The mean diagnostic delay, although decreasing, is 6 years, during which patients are therapeutically mismanaged [17]. Misdiagnosis is seen in 49% of people with CH most often with migraine, tension-type headache and sinusitis and removal of a healthy tooth has been reported in 15–43% [18–20]. Females are misdiagnosed more frequently than males with suggested reasons being pre-assumptions of women having migraine, a lower male:female ratio in chronic patients and differences in the clinical presentation [12, 13].

Self-rated health is strongly associated with mortality, making it an important instrument when investigating the burden of a disease [21]. Self-rated health is significantly reduced in ECH and in CCH the odds are tenfold lower of rating their health as ‘good’ or ‘very good’ compared to matched controls [15]. Co-existing depression and anxiety also occur more frequently in people with CH compared to controls [18, 22–24]. Suicidal thoughts are reported by 47–55% and attempts by 1.3–2% of people with CH [18, 24–26].

*“Although I’m not in bout, I still fear attacks every day”*

Growing evidence indicates that people with ECH are both physiologically and psychologically impacted even in remission periods, implying that ECH is also a chronically disabling disease despite the cyclic nature. Self-rated quality of life is significantly lower in people with ECH during remission compared with healthy controls [15] perhaps partly explained by worrying and avoidance behavior towards potential triggers [23] In addition, observational studies suggest that parameters such as sleep and other hypothalamic functions are altered not only during the active disease phase but also up to one year in remission [27–29] i.e., the attacks might be regarded as “the tip of the iceberg” in relation to pathophysiological activity.

An extensive epidemiological study from Sweden has shown that twice as many people with CH than age, sex and demographically matched controls were on disability pension (10.3% vs. 5.8%) [30] which is in agreement with previous studies [15, 18, 19, 30, 31]. The number of sick-days are also significantly higher for people with CH than controls [15, 31].

The economic burden of CH is significant and a call for attention [15, 32]. The direct costs of medication and healthcare services in a Danish study sum up to 5.178 euros per patient per year (CCH 9,158 euros), mainly due to acute medication and hospital admittance [15]. A German study from 2011 found direct costs per patient to be 4,737 euros for half a year [32] and including indirect costs the yearly costs amount to 11,739–11,926 euros per patient [15, 32]. Although not directly comparable (reports costs per bout), An Italian study found that the total cost of a CH bout was €4398 per patient and total cost of CCH was 5.4 times higher than ECH (€13,350) [33].

### From the perspective of a headache specialist

Treatment is initiated using a trial-and-error approach, and the close follow-up required is challenging in most health-care settings due to organizational and resource limitations. It is a significant limitation in CH treatment that existing guidelines are based on very few and small studies not fulfilling modern standards. They overall agree on first and second choice treatments but vary on recommended dosages and electrocardiogram (ECG) monitoring intervals. When exceeding the first- and second-line options, the evidence is even more sparse.

**Fig. 1:**
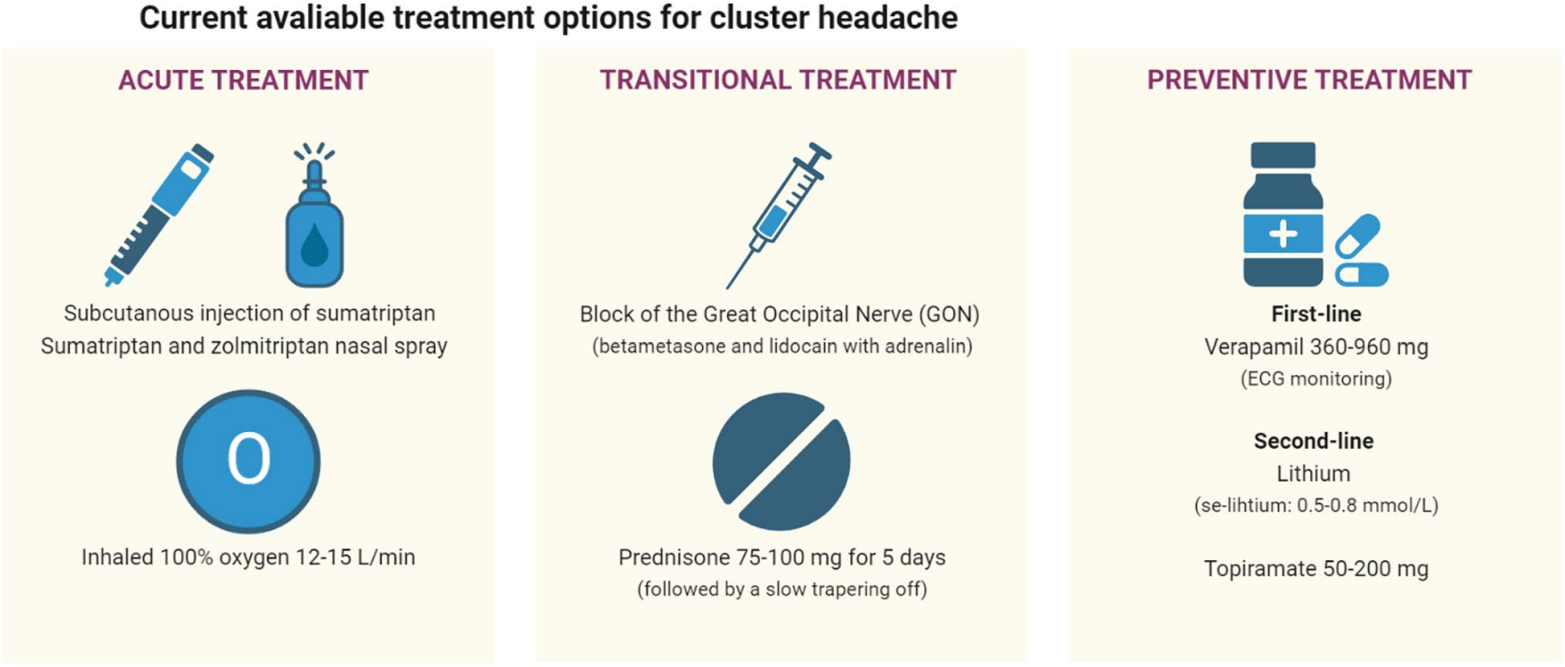
Current available treatment options for cluster headache

The treatment of CH can be divided into three categories 1) acute treatment aiming to abort the single attacks, 2) preventive treatment that taken at regular intervals aims to lower attack frequency and pain intensity, and lastly 3) transitional treatment that can be used as a short-lasting preventive if bouts are short or, more often, to obtain a “bridging” effect in the period a preventive is titrated to its therapeutic threshold (Fig. 1). The goal must always be to suppress attacks with preventives minimizing the need for acute treatment.

### Acute treatment

Treatment with 100% oxygen and triptans are the cornerstones of acute CH treatment and it is recommended to prescribe both. Simple analgesics and opioids are not effective [34]. In addition, an inappropriate use of opioids increases the risk of substance abuse.

#### Oxygen

One large randomized, double-blind, placebo-controlled crossover study with 109 participants showed that 78% inhaling 100% oxygen were pain-free or reported an adequate effect after 15 min compared to 20% receiving air [35]. These findings were confirmed in a small double-blinded cross-over study with 19 participants [36] and in an open-label study in 33 episodic- and 19 chronic participants [37]. An international survey covering 56 countries (23% of responders were from Europe) found that more than half of the participants reported use of oxygen in CH to be “very effective” or give “complete” remission [38]. Data from the Danish CH Survey similarly found that 75% had a 50% response to oxygen [8]. Oxygen is generally safe and without side-effects, however, it is unhandy to carry around and use multiple times a day outside of the residence. Furthermore, availability differs between countries. Oxygen is fully reimbursed (or with minor restrictions) in only 12 countries accounting for 63% of the European population [39].

#### Triptans

Triptans are easy to carry along, but they are costly and official guidelines limit the use to twice per day. However, based on an individual assessment and lack of options, many people with CH exceed this limit in agreement with their neurologist. Triptans are contraindicated in people with certain cardiovascular diseases as the vasoconstrictive effect has been theorized to increase the risk of stroke and acute myocardial infarction. Again, people may be so burdened that usage may still be offered after thorough information. The administration route affects efficacy. Subcutaneous injectable sumatriptan has shown to induce complete pain freedom in 20 min in 75% of participants; [40, 41] sumatriptan nasal spray induced pain freedom in 47% versus 18% for placebo at 30 min; [42] in episodic participants, oral zolmitriptan 10 mg induced meaningful pain reduction in 47% versus 29% for placebo; [43] and the effect of nasal zolmitriptan 5 and 10mg within 30 min was 40% and 62% [44]. Oral formula is generally not recommended due to its slower effect but may be the only available treatment in many countries. Triptans are reimbursed completely or with minor restrictions in 16 European countries, representing 66% of the population [39]. However, it is the authors’ experience that people on social support still find it difficult to pay for injectable/nasal sumatriptan.

Overall, oxygen and triptans are effective, however, the major problem lies with the high number of daily CH-attacks which necessitates too high (off-label) daily intake of triptans, with non-responders, patients with limited access to the medication (not available or too expensive), or patients with cardiovascular comorbidities. These people may end up with a problematic use of opioids or illegal drugs [26]. In pregnancy and during breastfeeding, treatment with oxygen is considered safe, recommendations on use of sumatriptan are varying, from limited use to no use [45].

### Preventive treatment

Preventive treatment is the cornerstone of CH management in order to suppress or limit the extreme pain attacks. Even for people with effective acute treatment the effect is not instant. Therefore, it is recommended, but not evidence based, that people with ECH start preventive treatment as soon as attacks are emerging and to slowly taper off after two weeks without attacks (allowing for swift increase again if attacks reemerge). In CCH there is need for continuous prevention.

The existing treatment recommendations are based on small and low level of evidence studies (listed in Table 1). This would not necessarily be a problem if clinical experience was that they were well tolerated and effectful, however, this is not the case. We will review the existing literature on the three major preventive treatment options in CH: verapamil, lithium and topiramate.

**Table 1:** RCTs on preventive treatment for cluster headache

Treatment	Type of study	Parti-cipants	Summary of efficacy	Effect	Clinical notes
**Verapamil**	Double-blind RCT from 2000 (360 mg/day)	30 eCH patients	2.week: 12/15 (80%) has > 50% reduction in attack frequency compared with 0% in the placebo group	Moderate	First choice
					ECT monitoring mandatory
	Study duration 19 days (5 days run in, 2 weeks treatment)				
			Mean attack frequency 0,6 in the verapamil group compared with 1,65 in the placebo group (p > 0,001)		
	Double-blind cross-over comparison between verapamil 360 mg/day and lithium 900 mg/day from 1990	30 cCH patients	1^st^ week of treatment: 50% on verapamil had a reduction in a non-specified headache index		
	Study duration 23 weeks in total (Two 8 week-treatment periods without wash-out)				
**Lithium**	Double-blind RCT from 1997 (800 mg/day)	27 eCH patients	After 1 week no difference between groups (2/14 on lithium and 2/13 on placebo had cessation of attacks)	Moderate	Not suitable for short bouts and patients with poor compliance
	Study duration 7 days				
					ECG monitoring and blood samples mandatory
	Double-blind cross-over comparison between verapamil 360 mg/day and lithium 900 mg/day from 1990 Study duration 23 weeks in total ( 2 8 week treatment periods without wash-out)	30 cCH patients	1^st^ week of treatment: 50% on verapamil had a reduction in a non-specified headache index		
**Topiramate**	No RCTs			Inconclusive	Monitor mood changes
**cGRP antibody Galcanezumab**	Double-blind multicenter RCT from 2019 (s.c.300 mg/month) Study duration: a prospective baseline period and an 8 week double-blind, placebo-controlled period	106 eCH patients were randomized	50% attack reduction in 71% patients receiving galcanezumab compared to 53% receiving placebo Mean weekly attack frequency across weeks 1 through 3 was 8.7 for galcanezumab and 5.2 for placebo (p = 0,04)	Moderate	Very few side effects and contra indications
	Double-blind multicenter RCT from 2020 (s.c. 300 mg/month) Study duration: A prospective baseline period, a 12-week double-blind, placebo-controlled treatment period, and a 52-week open-label period	237 cCH were randomized	Mean change in weekly attack frequency was 4.6 placebo versus 5.4 galcanezumab (p = 0.334)	Not effective /Inconclu-sive (study design not optimal)	Very few side effects and contra indications Study halted before final inclusion

#### Verapamil

The understanding of its mechanism of action in CH remains unclear. Among the suggested are vasospasm inhibition [46], GABA-A inhibition [47, 48], circadian rhythm modulation [49, 50], and hyperpolarization-activated cyclic nucleotide-gated channel mediated decreasing of parasympathetic activity [51].

The rationale for using verapamil as a first-line preventive treatment is based on two randomized controlled trials (RCTs) and three open label studies. The first double-blind cross-over RCT lasted 23 weeks comparing verapamil 360 mg/day with lithium 900 mg/day in CCH (no placebo group). Only 50% on verapamil and 37% on lithium experienced a reduction in an unspecified headache index [52]. It remains unknown what the index included, however, the study established the foundation for verapamil treatment. The second study randomized 30 people with ECH to either 14 days with verapamil 360 mg/day or placebo. In the second week, 80% on verapamil reported 50% or higher reduction in attack frequency compared with 0% on placebo, however, just 27% became attack free [53].

An open-label study from 1983 tested several potential drug targets, including verapamil 160–720 mg/day finding an unspecified effect in five participants with CCH [46]. This was followed by another open-label study from 1989 with 33 episodic- and 15 chronic participants receiving verapamil 240–1200 mg/day. Improvement was seen after an average of 1.7 weeks and 5 weeks for ECH and CCH, respectively. As many as 69% reported a more than 75% effect [54]. In the largest open-label study of 52 episodic- and 18 chronic participants, the authors aimed to personalize timing and dosage of verapamil. With verapamil 360–920 mg/day 94% episodic- and 56% chronic participants became attack free. Based on previous bout length and intensifying clinical presentation, people with ECH were included if expected bout length exceeded a few days [55]. The major limitation of the open label studies is the lack of a control group as the level of placebo response and spontaneous remission is unknown. For CH there is an extreme variability in bout length from bout to bout and as many as 20% may change phenotype over time [56].

In sharp contrast to these reports, new data from 400 consecutively recruited Danish persons with CH show that only 44% episodic- and 34% chronic participants reported a reduction in attack frequency more than 50%. Only 14% reported complete relief of attacks on verapamil [8]. Efficacy does not seem to be associated with high/low dose or sex, but verapamil is more effective in ECH compared to CCH [50, 57]. Side effects such as constipation, tiredness and oedema are reported in 12–86% of participants [46, 52–55]. Overall, it is the authors’ experience, that people with CH are willing to accept substantial side effects if they experience some relief. Also, we will dispute that most have side effects on effective dosages of verapamil, but only very limited evidence of this is available.

A whole range of cardiac contra-indications exist for verapamil including untreated 2^nd^ and 3^rd^ degree atrioventricular block, bradycardia and heart failure, severe hypotension, and Wolff-Parkinson-White-Syndrome. In addition, the list of possible interactions is long including frequently used medications as atorvastatin (increased risk of myopathy and rhabdomyolysis), domperidone (risk of prolonged QT interval), clopidogrel (decreased antiplatelet effect), fluconazole (increased verapamil exposure) and lithium (neurotoxicity and bradycardia) [58, 59].

#### Lithium

Lithium is recommended as second line treatment, but is most suited for people with CCH and has several limitations, discussed in the following section. Slowly titrating to a dose of 600-1500mg daily with serum levels between 0.6 and 0.8 is considered optimal. It is speculated that treatment with lithium was initiated due to the cyclic nature of the disease (as is the case for bipolar disease). Only two randomized controlled studies exist, one showing no effect in 27 episodic participants (note only 1 week of treatment) [60] and one comparing lithium treatment with verapamil showing that 37% of chronic participants experienced a reduction in an unspecified CH index [52]. The effect of lithium has been reviewed since 1981 where several open label studies indicated a good effect, particularly in CCH [61].

The main problem with lithium is the high degree of acute and long-term side effects and that treatment requires frequent blood samples to titrate the dosage according to the therapeutic index, to avoid toxic serum levels and to control for adverse effects on kidney, liver and thyroid function [5, 7]. This is burdensome for both the patient and the health-care system.

#### Topiramate

Is also recommended as second line treatment and may be used in both ECH and CCH. The anticonvulsant drug is recommended in doses of 50–200 mg daily by the 2019 EHF guidelines [10]. The preventive effect of topiramate has been investigated in two small open label studies with 13 and 33 participants with conflicting results. The largest study found more than 50% reduction in only 21% of participants (study period of 20 days) and the smallest showed that 75%, mostly episodic participants, went into remission [62, 63]. The benefit of topiramate is that cardiac monitoring is not required. However, depression is a known side-effect; especially in people with pre-existing depressive symptoms, which is reported in up to 67% of people with CH [24]. Other prominent side-effects, often leading to discontinuation in the clinic, are cognitive impairment and paresthesia. Topiramate cannot be used in people with kidney stones.

#### Other preventive options

In treatment refractory patients, it may be necessary to try medical treatment with even lower level of evidence either as monotherapy or as add-on. Treatment with 10 mg oral melatonin was shown effective in a small RCT in 5 out of 10 people with ECH compared to none in the placebo group [64]. In a small case–control study, mainly with chronic patients, no effect was observed [65]. OnabotulinumtoxinA may have some additional effect in treatment refractory CCH [66], however, evidence is sparce and the pathophysiological mechanism behind an effect using a migraine protocol is uncertain. Although not available in most European countries, it is worth to mention that retrospective chart review has suggested a possible effect of short-term (3–5 days in hospital) intravenous treatment of dihydroergotamine [67] and 1–2 mg ergotamine without caffeine given at night may also prevent nightly attacks and nausea prevented in advance [9].

#### LSD and Psilocybin

Use of illicit drugs like psilocybin, lysergic acid diethylamide (LSD) and gamma-hydroxybutyrat (GHB) are more frequently reported by people with CH compared to the general population [64]. Several retrospective surveys and case reports indicate that psilocybin and LSD/the non-halluzinogenic bromo-LSD in some cases may abort attacks and extend the duration of remission periods [65, 66]. An explorative RCT with 17 episodic and chronic participants investigated microdosing of psilocybin finding no difference between groups in efficacy and side effects [67]. As with other medical treatment, RCTs are needed to evaluate efficacy and safety before treatment can be recommended.

### Transitional treatment

Current preventive medications need to be titrated up to an effective dosage, and an intermediate treatment consisting of corticosteroids can therefore be applied if patients are burdened by many attacks [10]. The European 2019 EHF guidelines define treatment with either prednisone taken orally or given as a greater occipital nerve block as intermediate treatment, whereas the 2023 European Academy guidelines and the 2006 EFNS guidelines include them under preventive treatment [9–11].

#### Prednisone

The exact effect mechanism is poorly understood, but oral corticosteroids have been suggested to attenuate trigeminal activation and counteract hypothalamic dysfunction [68]. A multi-centre, double-blind, RCT from 2021 showed a fast onset of 100 mg prednisone in 118 episodic participants with 7.1 attacks compared with 9.5 in the placebo group within the first week [69]. Two studies from 1978 and 1975; the first a case-series in 19 participants showed that 58% became attack free with 10–80 mg prednisone daily for 3–10 days [70] and the second, a double-blind single cross-over study, also indicated efficacy [71]. Short-term use is considered effective and safe (although people on rare occasions may develop psychiatric symptoms), but continuous use may increase the risk of known systemic side effects of prednisone (opportunistic infections, hypertension, osteoporosis and metabolic diseases such as type 2 diabetes).

### Greater occipital nerve (GON) blocks

The effect is thought to occur through a modulatory effect on the nociceptive processing in trigeminal neurons via the trigemino-vascular system [5]. Two double-blind RCTs exist. The first investigated three injections of cortivazol in 1 week in 28 episodic- and 15 chronic participants. Two to four days after the third injection, 95% in the active group had two or less daily attacks compared to 55% in the placebo group. Attack frequency was also reduced to one third of that in the placebo group in the first 15 days [72]. Attack-freedom was seen in 85% of 16 episodic- and 7 chronic participants one week after a single dosage of betamethasone compared with none receiving placebo [73]. GON blocks has shown higher efficacy in episodic participants compared to chronic in a prospective open-label study [74]. Most clinics use 2.5 mL betamethasone (rapid and long acting) plus 0.5 mL lidocaine 2% sc ipsilateral to the pain.

Side effects of short term and long-term use are equal to oral use. Having this in mind, in the represented clinics of this paper, injections in 3 months intervals are considered safe. Repeated nerve blocks in medically refractory people with CCH led to transient attack freedom in only one third [75]. GON blocks are generally accepted for usage in pregnant and breastfeeding women [45].

### Oral Triptans with longer half-live time

Although there is no evidence from clinical trials, it is the authors clinical experience that frovatriptan and naratriptan may be used for transitional prophylaxis in cases where GON blocks are inefficient or contraindicated or as a short term mini-preventive in people with several nightly attacks and short bouts [76–78].

### From the perspective of a cardiologist

People with CH have a high burden of cardio- and cerebrovascular (CVD) risk factors, including high body mass index (for males) and smoking, which is reported by 48–68% of patients in recent publications [12, 22, 79, 80]. These factors are known to increase the risk of CVD. Cross-sectional studies have shown that overall multimorbidity including CVD occur more frequently in people with CH than in matched controls [14, 22]. Therefore, use of triptans, verapamil and lithium may be worrisome.

Triptans have an extracranial vasoconstrictive effect and are relatively contraindicated in people with known CVD. Although prescribers seem to take this into account, a novel Italian study showed that 4% of male patients were treated with triptans despite having a CVD [81]. Retrospective data on CH patients with more than two daily dosages have not reported serious adverse events, however official guidelines still limit the daily use to two. With a daily attack frequency of up to eight, triptans can seldomly stand alone.

As discussed above, verapamil is the first-line preventive medication. The highest recommended daily dose in cardiology is 480 mg and combination with betablockers are not recommended due to risk of atrioventricular block. ECG should be assessed before initiation -*each time* and before increasing above 400 mg, 600 mg, 800 mg and 1000 mg. In patients treated with higher dosages than 480 mg, an annual ECG is recommended and in case of sinus bradycardia, 1. degree AV block or symptoms as syncope, fatigue or dizziness, Holter should be performed. From clinical experience, patients often forget ECG controls when initiating treatment when a new bout begin or if increasing the dosage. This concerning issue is also the finding in audit data on 217 English CH participants on verapamil showed that 41% received verapamil treatment without an ECG and among those with, 19% had arrhythmias, with prolonged PR interval being the most frequent [82].

Lithium is known to induce benign ECG alterations and near fatal arrhythmias but may also have cardioprotective potential. At therapeutic lithium levels, T-wave depressions and sinus node dysfunction are the most common ECG findings. Arrhythmias are mainly noticed with high serum lithium. A baseline ECG is recommended and in case of elevated serum lithium levels or symptoms of arrhythmias, a novel ECG or Holter monitoring is needed.

### Future perspectives

An era of new specifically targeted treatments with few side effects are emerging in the headache field.

#### Anti-cGRP therapy

cGRP antibodies are the first targeted medical treatment possibilities in CH based on a pathophysiological understanding of the disease [83]. CGRP plasma levels increases during spontaneous and nitroglycerin induced attacks and were reduced to baseline levels after spontaneous, sumatriptan- and oxygen-induced termination [84–86]. Further, a double-blind RCT found that cGRP infusion triggered attacks in people with chronic and active episodic CH, but not in remission [87]. In a phase III RCT, a 50% attack reduction was seen in 71% of episodic participants treated with galcanezumab vs. 53% treated with placebo and mean weekly attack frequency across weeks 1 through 3 was significantly reduced with 40% [88]. Of most importance, cGRP antibodies are generally very well tolerated with few side effects. On this basis, galcanezumab was approved for the treatment of ECH in the US and Canada but not in Europe as the European Medical Agency found effect and evidence to be too sparce [89]. A more recent Korean open label study on 240 mg galcanezumab in ECH supports the findings of the RCT [90]. Galcanezumab did not meet the primary or secondary efficacy endpoint in CCH [91]. Studies on fremanezumab in ECH and CCH were aborted, as futility analyses concluded that the primary endpoints were unlikely to be met [92, 93] and the recent study in ECH with eptinezumab has stopped further inclusion after futility analyses. There is an ongoing open label trial with eptimezumab for CCH [94] and erenumab for CCH [95]. Recently, recommendations on optimal RCT design in ECH and CCH have emerged [96].

#### Neuromodulation and invasive procedures

Neuromodulation has become an emerging and viable treatment option for medically treatment refractory CCH patients e.g. treatment failure of three preventive drugs [97]. Despite being restricted to a minority, invasive and very costly, neurostimulation greatly reduces patient burden and subsequently both indirect and direct healthcare costs [98]. In extremely severe cases, deep brain stimulation has been described in case series, but proper trials into efficacy, safety and the optimal stimulation target are lacking [99, 100]. After several case series [101–107] the ICON (intractable chronic cluster headache) trial provided evidence for the efficacy of occipital nerve stimulation (ONS) in an international, multicenter phase 3 RCT. In the 131 chronic participants, mean attack frequency was reduced from 15.8 weekly attacks to 7.4 during the one-year study period for both high and low electrical dose. ONS is now reimbursed for medically intractable CCH in several European countries.

There are two RCTs investigating sphenopalatine ganglion stimulation (SPG) versus sham stimulation in CCH as acute treatment finding a 10% difference achieving pain-freedom in 15 min versus sham [108, 109]. Long-term open label studies found that 33% experienced a preventive effect and that 78% of attacks were successfully treated with SPG and 74% of participants with CCH could reduce or remain off all preventive medication when using SPG stimulation, [110, 111] however treatment is currently unavailable [112]. Navigation guided botulinumtoxin injections targeting the SPG is currently being investigated in a multinational RCT [113] as pilot data have indicated safety and efficacy in CCH [114].

There are three randomized trials assessing vagus nerve stimulation (NVS) as both acute and preventive treatment [115–117]. As acute treatment, there was no difference in pain freedom after 15 min between NVS and sham. For prevention, the open-label study in CCH found a significant reduction of 4 weekly attacks in the NVS group versus sham. The 50% responder rate was 40% in the NVS versus 8% in the sham group [115]. NVS seems a viable but fairly costly option in those patients who are unresponsive or have a contraindication against triptans.

## Conclusion

As long as people with CH have to endure and fear CH attacks, the impact on their lives and the associated societal burden remains enormous. Effective preventive medication that can be taken as soon as attacks emerge, with a rapid onset of effect and few side effects must be the ultimate goal when treating CH. New preventive treatment, investigated according to modern standards and of high quality, are needed. They may be more expensive but at present the major costs are due to acute medication and hospital admissions. With an effective preventive treatment these costs are expected to be greatly reduced, adding to overall cost-effectiveness. Other challenges are accessibility to existing acute and preventive treatment, increasing knowledge of CH in the general population and general practitioner to obtain better social support and not least to secure a smoother diagnostic process.

## Data Availability

Not applicable.

## Abbreviations

CCH: Chronic cluster headache
CGRP: Calcitonin gene-related peptide
CH: Cluster headache
CVD: Cardiovascular disease
ECG: Electrocardiogram
ECH: Episodic cluster headache
EFNS: European Federation of Neurological Societies
EHF: European Headache Federations
GHB: Gamma-hydroxybutyrat
GON: Greater occipital nerve
LSD: Lysergic acid diethylamide
NVS: Vagus nerve stimulation
RCT: Randomized clinical trial
SPG: Sphenopalatine ganglion
